# Identification of the Shared Gene Signatures and Biological Mechanism in Type 2 Diabetes and Pancreatic Cancer

**DOI:** 10.3389/fendo.2022.847760

**Published:** 2022-03-31

**Authors:** Yifang Hu, Ni Zeng, Yaoqi Ge, Dan Wang, Xiaoxuan Qin, Wensong Zhang, Feng Jiang, Yun Liu

**Affiliations:** ^1^ Department of Geriatric Endocrinology, The First Affiliated Hospital of Nanjing Medical University, Nanjing, China; ^2^ Department of Dermatology, Affiliated Hospital of Zunyi Medical University, Zunyi, China; ^3^ Department of Neurology, The First Affiliated Hospital of Nanjing Medical University, Nanjing, China; ^4^ Department of Pharmacy, The First Affiliated Hospital of Nanjing Medical University, Nanjing, China; ^5^ Department of Neonatology, Obstetrics and Gynecology Hospital of Fudan University, Shanghai, China; ^6^ Department of Medical Informatics, School of Biomedical Engineering and Informatics, Nanjing Medical University, Nanjing, China

**Keywords:** pancreatic cancer, type 2 diabetes, WGCNA, endodermal cell fate specification, S100A6

## Abstract

**Background:**

The relationship between pancreatic cancer (PC) and type 2 diabetes mellitus (T2DM) has long been widely recognized, but the interaction mechanisms are still unknown. This study was aimed to investigate the shared gene signatures and molecular processes between PC and T2DM.

**Methods:**

The Gene Expression Omnibus (GEO) database was used to retrieve the RNA sequence and patient information of PC and T2DM. Weighted gene co-expression network analysis (WGCNA) was performed to discover a co-expression network associated with PC and T2DM. Enrichment analysis of shared genes present in PC and T2DM was performed by ClueGO software. These results were validated in the other four cohorts based on differential gene analysis. The predictive significance of S100A6 in PC was evaluated using univariate and multivariate Cox analyses, as well as Kaplan–Meier plots. The biological process of S100A6 enrichment in PC was detected using Gene Set Enrichment Analysis (GSEA). The involvement of S100A6 in the tumor immune microenvironment (TIME) was assessed by CIBERSORT. *In vitro* assays were used to further confirm the function of S100A6 in PC.

**Results:**

WGCNA recognized three major modules for T2DM and two major modules for PC. There were 44 shared genes identified for PC and T2DM, and Gene Ontology (GO) analysis showed that regulation of endodermal cell fate specification was primarily enriched. In addition, a key shared gene *S100A6* was derived in the validation tests. S100A6 was shown to be highly expressed in PC compared to non-tumor tissues. PC patients with high S100A6 expression had worse overall survival (OS) than those with low expression. GSEA revealed that S100A6 is involved in cancer-related pathways and glycometabolism-related pathways. There is a strong relationship between S100A6 and TIME. *In vitro* functional assays showed that S100A6 helped to induce the PC cells’ proliferation and migration. We also proposed a diagram of common mechanisms of PC and T2DM.

**Conclusions:**

This study firstly revealed that the regulation of endodermal cell fate specification may be common pathogenesis of PC and T2DM and identified S100A6 as a possible biomarker and therapeutic target for PC and T2DM patients.

## Introduction

Pancreatic cancer (PC) is the third most deadly disease among all malignancies in the USA, and more deaths are expected to occur in the coming years (1, 2). The pancreas contains both exocrine and endocrine parts, and approximately 95% of PC originates from exocrine cells. The most common type of PC is pancreatic ductal adenocarcinoma (PDAC), with a 5-year survival of <10% estimated by the American Cancer Society in 2020 (1). The high mortality is mostly since about 85% of PC patients are usually diagnosed at an advanced age when cancer has metastasized and spread, making great difficulties for surgery (3). Early detection, as well as early treatment of PC, is therefore of utmost importance.

It is necessary to identify individuals at a high risk of developing PC. Numerous studies have specified type 2 diabetes mellitus (T2DM) as a known risk factor for PC (4, 5). T2DM is the most common endocrine disease, characterized by insulin resistance and pancreatic β-cell malfunction. Initially, to compensate for insulin resistance *in vivo*, more insulin is secreted by the endocrine pancreas to enhance peripheral glucose utilization. With time, the continuous metabolic stress ultimately leads to β-cell’s progressive failure and decreased function (6). In the pathogenesis of T2DM, insulin released from pancreatic β-cells into the intrapancreatic portal circulation nourishes acinar and ductal cells adjacent to the islets, thus promoting their proliferation and increased risk of PC (7). On the other hand, some researchers believe that diabetes is also a consequence of PC (8). Possible mechanisms include hyperinsulinemia in PC, PC-induced insulin resistance, impaired β-cell, and impaired insulin secretion (9). Therefore, the association of T2DM and PC presented a “causal” relationship in the course of the disease. Although there is strong clinical and epidemiological evidence on the link between PC and T2DM (10–13), little is known about their shared signatures based on gene regulation mechanisms.

Recently, advances in sequencing technology and bioinformatics have made it possible to explore the common pathogenesis of disease–disease interaction at the genetic level (14, 15). The present study aimed to discover co-expression clusters and shared genes of PC and T2DM using the weighted gene co-expression network analysis (WGCNA). Our results revealed that PC and T2DM were shown to share 44 genes in test cohorts that were mainly enriched in the regulation of endodermal cell fate specification, which may be considered as a common mechanism of PC and T2DM. In validation cohorts, the discovery of a hub shared gene, *S100A6*, in both PC and T2DM was particularly noteworthy, which may serve as a predictive biomarker for detecting covert PC in T2DM patients.

## Materials and Methods

### Dataset Download and Process

We used the term “type 2 diabetes mellitus” or “pancreatic cancer” to search for T2DM and PC patients’ gene expression profiles in Gene Expression Omnibus (GEO) (http://www.ncbi.nlm.nih.gov/geo/) database. The obtained datasets were screened by the following criteria. First, profile information should include both case and control groups. Second, all samples should be derived from pancreas tissue. Third, pancreatic cancer was not treated with chemoradiotherapy before surgical resection. Fourth, these datasets must provide raw data that can be further analyzed. Last, GEO datasets, GSE38642, GSE20966, GSE25724, GSE91035, GSE55643, and GSE32688, were chosen for next research. In addition, the somatic mutation data and transcriptome data were acquired from The Cancer Genome Atlas (TCGA) (https://portal.gdc.cancer.gov/) database. HTSeq-FPKM value needs to be transformed into transcripts per million (TPM).

### Weighted Gene Co-Expression Network Analysis

WGCNA is an algorithm for analyzing gene expression patterns in numerous samples. It is capable of clustering genes and constructing modules based on similar gene expression patterns, as well as analyzing the associations between modules and biological traits (16). In this study, we used the R package “WGCNA” to create the gene co-expression networks of T2DM and PC. Firstly, we built the adjacency matrix using soft-threshold b (7 for T2DM, 8 for PC) and gene–gene correlation matrix to describe the degree of association between the nodes. The adjacency matrix was then converted into the topological overlap matrix (TOM). Next, the gene hierarchical clustering dendrogram was performed to identify co-expression modules. Finally, we calculated the module eigengene (ME), as well as the correlation between ME and clinical traits, to identify clinical-related modules.

### Detection of Shared Genes in Type 2 Diabetes Mellitus and Pancreatic Cancer

The shared genes in T2DM and PC modules with positive correlation coefficients were overlapped using Jvenn (17). ClueGO, a plug-in of Cytoscape, could classify non-redundant GO terms and visualize the functionally related genes in a clustered network (18). To understand the biological function of these shared genes, we performed a GO analysis by utilizing the ClueGO. The p-value <0.05 was identified as the significant GO term.

To verify the shared gene signatures in T2DM and PC, the protein–protein interaction (PPI) network was next created using the “MCODE” algorithm with default settings in Cytoscape. The hub clusters were further enriched by GO. Disease Ontology (DO) analysis was conducted using R package “DOSE” (19).

### Validation of Shared Genes in Type 2 Diabetes Mellitus and Pancreatic Cancer

To confirm hub shared genes in T2DM and PC, we conducted the differentially expressed gene (DEG) analysis on validation datasets (GSE20966, GSE25724, GSE55643, and GSE32688) using the R package “limma.” p < 0.05 was considered as the cutoff value. The overlap of above shared genes in test cohorts and DEGs in validation cohorts was represented by a Venn diagram using Jvenn tool.

### Gene Set Enrichment Analysis

Based on S100A6 median expression, PC patients were categorized into high- and low-expression groups. GSEA is a method based on functional categories that could calculate the enrichment score of gene sets and discover different functional phenotypes (20). We used GSEA to compare the biological pathways between the two groups. The h.all.v7.2.symbols.gmt gene set was downloaded as a reference. Kyoto Encyclopedia of Genes and Genomes (KEGG) significant pathways with false discovery rate (FDR) <0.05 were included.

### Assessment of the Immune Landscape

The tumor immune microenvironment (TIME) is assessed by single-sample Gene Set Enrichment Analysis (ssGSEA) method using R package “GSVA.” ssGSEA, an extension of GSEA, could calculate separate enrichment scores (ESs) for each sample (21). Geneset variation analysis (GSVA) is an algorithm that converts an expression matrix with a single gene into an expression matrix with a specific gene set and calculates its ES (22). The levels of 16 immune cells and 13 immune-related functions between high- and low-S100A6 expression groups were then compared by ssGSEA scores. ESTIMATE is a tool to compute each sample’s immune score and stromal scores (23). Spearman’s correlation was used to investigate the relationship between S100A6 and immune cell infiltrates.

### Cell Culture and Transfection

The PC cells were cultured in Dulbecco’s Modified Eagle Medium (DMEM) with 10% Fetal bovine serum (FBS) at 37°C in a 5% CO_2_ incubator. The cells in logarithmic phase were chosen for functional experiments. S100A6 knockdown was achieved by transfecting cells with indicated S100A6-siRNA using Lipofectamine^®^ 3000 reagent (Invitrogen, USA) as directed by the manufacturer’s instructions. The target sequences for siS100A6-1 and siS100A6‐2 were 5’-GCUCACCAUUGGUGCUAAG-3′ and 5′-CCUCUCUGAGUCAAAUCCATT-3′.

### RNA Extraction and qRT-PCR

Total RNA was isolated from cells using TRIzol reagent (Invitrogen, America), and cDNAs were generated using HiScript Synthesis kit (Vazyme, China). Next, real‐time quantitative PCR (qRT-PCR) analysis was carried out using the Fast SYBR Green Master Mix (Roche, America) on the StepOnePlus Real-Time PCR system (Applied Biosystems, CA, USA). Primers were as follows: S100A6: forward, 5’- GGGAGGGTGACAAGCACAC-3’; reverse, 5’- AGCTTCGAGCCAATGGTGAG-3’.

### Cell Counting Kit-8 and Colony Formation

Cell counting kit-8 (CCK-8) and colony formation were conducted to evaluate PC cells’ proliferative abilities. For CCK-8 assay, cells were seeded at 2,000 cells per well in 96-well plates overnight; cell growth was then monitored at various time points using the CCK-8 kit (C0038, Beyotime, China), and 2-h absorbance at 450 nm was measured on an enzyme labeling. For colony formation, about 1,000 cells were placed into each well in six-well plates. When colonies appeared, 4% methanol and crystal violet were successively used to fix and stain the cells.

### Transwell Invasion Assay

The 24-well Transwell chambers precoated with Matrigel (Corning, USA) were used to perform invasion assays. About 2 × 10^4^ MIA PaCa-2 cells were seeded into the upper chambers of the Transwell in serum-free DMEM medium, while the lower chamber was supplemented with DMEM containing 10% FBS. After 24 h, the penetrated cells were fixed with 4% methanol, then stained with crystal violet, and finally photographed and counted under an inverted microscope.

## Results

### Dataset Information

A total of six GEO datasets numbered GSE38642, GSE20966, GSE25724, GSE91035, GSE55643, and GSE32688 were selected in this study; details were summarized in **Table 1**. We coupled the GSE38642 and GSE91053 as discovery cohorts for the WGCNA analysis and the remaining sets as validation cohorts. TCGA database was used for copy number variation (CNV) analysis.

**Table 1:** Information of GEO datasets containing the T2DM/PC patients.

ID	GSE number	Platform	Samples	Disease	Group
1	GSE38642	GPL6244	9 patients and 54 controls	T2DM	Discovery
2	GSE91035	GPL22763	25 patients and 23 controls	PC	Discovery
3	GSE20966	GPL1352	10 patients and 10 controls	T2DM	Validation
4	GSE25724	GPL96	6 patients and 7 controls	T2DM	Validation
5	GSE32688	GPL570	25 patients and 7 controls	PC	Validation
6	GSE55643	GPL6480	45 patients and 8 controls	PC	Validation

T2DM, type 2 diabetes mellitus; PC, pancreatic cancer; GEO, Gene Expression Omnibus.

### Co-Expression Modules in Type 2 Diabetes Mellitus and Pancreatic Cancer

**Figure 1:**
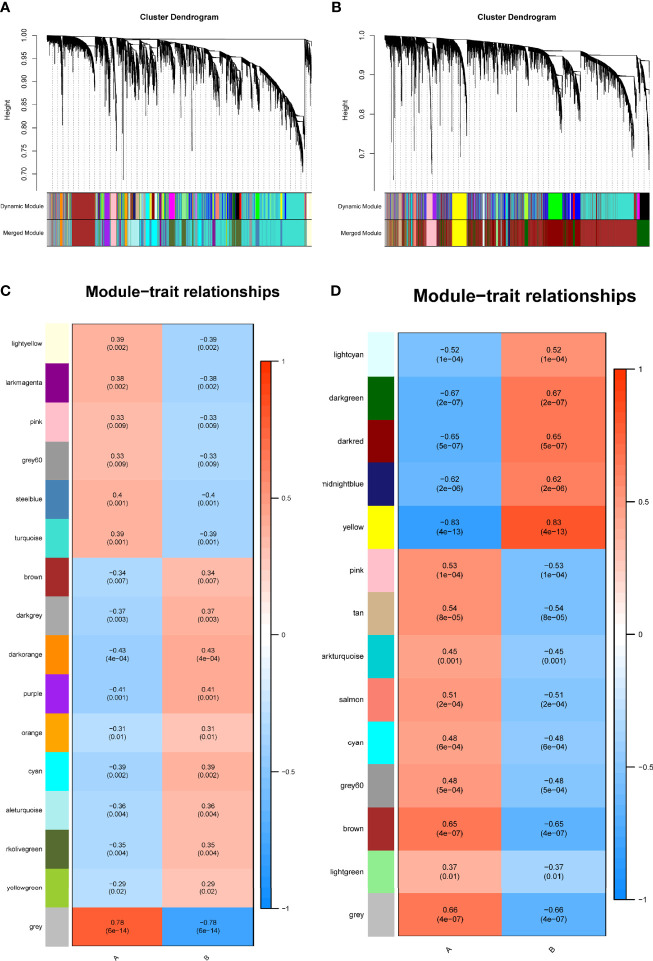
Identification of modules linked to clinical features of T2DM and PC by WGCNA. **(A, B)** Cluster dendrogram of co-expressed genes in T2DM **(A)** and PC **(B)**. **(C, D)** Heap of module–trait relationships in T2DM **(C)** and PC **(D)**. T2DM, type 2 diabetes mellitus; PC, pancreatic cancer; WGCNA, weighted gene co-expression network analysis.

We identified 16 modules in GSE38642 by WGCNA, and each module is represented by a different color. Based on Spearman correlation coefficient, a heat map about module–trait relationships was drawn to assess the relationships between modules (**Figures 1A, C**). Three modules “grey,” “darkorange,” and “purple” had a highly positive association with T2DM, which were chosen as T2DM-related modules (darkorange module: r = 0.43, p = 4e−04, genes = 55; purple module: r = 0.41, p = 0.001, genes = 107; cyan module: r = 0.39, p = 0.002, genes = 290). Additionally, 14 modules were discovered in GSE91035, with the module “yellow” (r = 0.83, p = 4e−13, genes = 444) and “darkgreen” (r = 0.67, p = 2e−07, genes = 1037) being highly positively linked with PC (**Figures 1B, D**).

### The Shared Genes in Type 2 Diabetes Mellitus and Pancreatic Cancer

**Figure 2:**
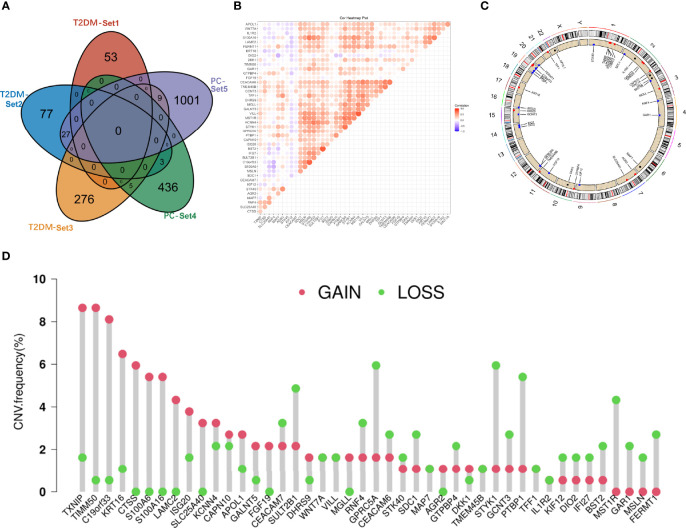
Characterization of the shared genes in T2DM and PC. **(A)** Venn diagram of the shared genes between the three T2DM modules and two PC modules. **(B)** Spearman correlation analysis of 44 shared genes of T2DM and PC in TCGA databases. **(C)** Location of CNV alteration of the shared genes on chromosomes. **(D)** CNV frequency of the shared genes in T2DM and PC. T2DM, type 2 diabetes mellitus; PC, pancreatic cancer; TCGA, The Cancer Genome Atlas; CNV, copy number variation.

T2DM and PC shared 44 genes in positivity related modules (**Figure 2A**), which were considered to be extremely associated with the pathogenesis of T2DM and PC. Most of these shared genes were correlated with each other and showed a strong and significant degree of correlation in TCGA database (**Figure 2B**). Interestingly, we also found CNVs in all 44 shared genes in TCGA PC database. We observed the alterations of these genes with CNVs on the chromosome (**Figure 2C**). There were 19 CNVs that were copy number gain and 25 CNVs that were copy number loss (**Figure 2D**).

**Figure 3:**
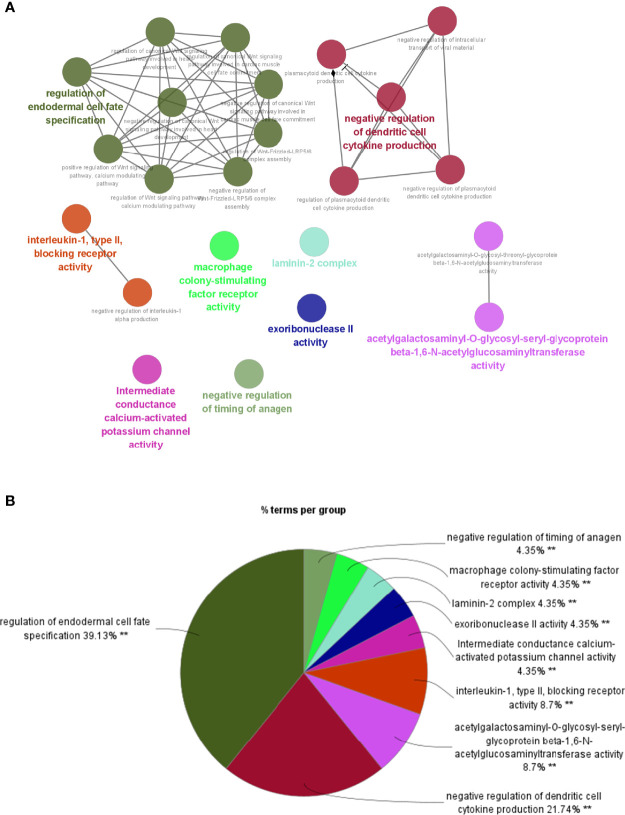
GO analysis of the shared genes in T2DM and PC. **(A)** The network of GO terms in ClueGO. **(B)** The percentage of GO terms in the shared genes. T2DM, type 2 diabetes mellitus; PC, pancreatic cancer; GO, Gene Ontology. ^**^p < 0.01.

**Figure 4:**
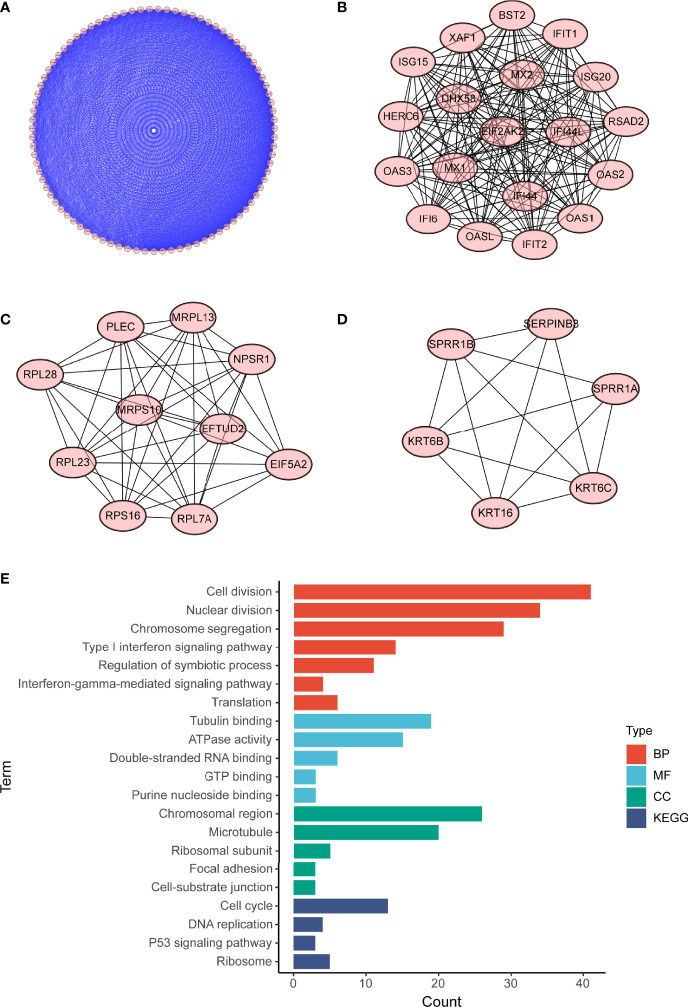
PPI network of PC Set5 module genes. **(A–D)** Four PPI clusters derived from PC Set5 module. **(E)** GO biological process of the four clusters. PPI, protein–protein interaction; PC, pancreatic cancer; GO, Gene Ontology.

To investigate potential roles of the shared genes in T2DM and PC, we used GlueGo to run a GO analysis. The results revealed that these genes were enriched in various biological activities including the regulation of endodermal cell fate specification, regulation of dendritic cell, Interleukin (IL)-1β-related signaling pathway, ion channel activities, and regulation of timing of anagen. Regulation of endodermal cell fate specification accounted for about 40% of total GO terms (**Figures 3A, B**), indicating great importance of this pathway both in T2DM and PC. The Set4 and Set5 modules were shown to be strongly related to PC, and the genes shared with T2DM were primarily found in Set5 module. Therefore, we continued to construct the PPI network at protein level from Set5 module. **Figures 4A–D** displayed four clusters by MCODE method (**Figures 4A–D**). Enrichment analysis suggested that they were mostly involved in pathways of cell division, cell cycle, nuclear division, DNA replication, transcription, translation, and symbiotic process (**Figure 4E**). These results were also visualized with a GO circle diagram (**Figure S1**). DO analysis showed that diseases enriched by the shared genes were linked with T2DM and PC (**Figure S2**).

### Hub Shared Gene S100A6 and Prognostic Value in Pancreatic Cancer

**Figure 5:**
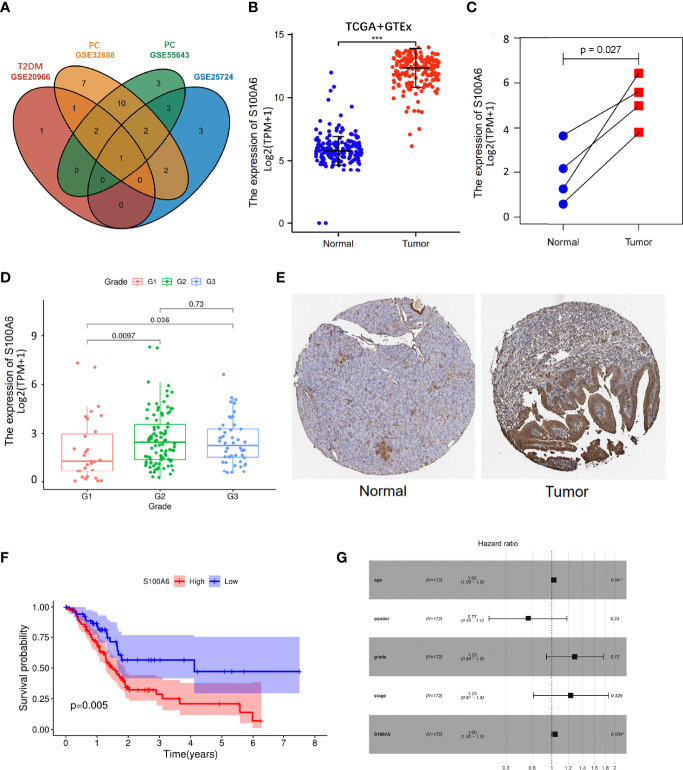
S100A6 expression and prognostic value in PC tissues. **(A)** Venn diagram of the shared genes in T2DM and PC in validation cohorts. **(B, C)** The expression levels of S100A6 in normal and PC tissues. **(E)** Representative IHC staining of S100A6 in normal and PC tissues from HPA database. **(F)** K-M curve of association of S100A6 and PC patients’ OS in TCGA cohort. **(G)** Multivariate Cox analysis of prognostic variables in PC patients. T2DM, type 2 diabetes mellitus; PC, pancreatic cancer; IHC, immunohistochemistry; K-M, Kaplan–Meier; OS, overall survival. ^∗^p < 0.05 and ^∗∗∗^p < 0.001.

To validate our findings, we conducted differential genes analysis between cases and controls on validation cohorts (GSE20966, GSE25724, GSE55643, and GSE32688). The shared genes in T2DM and PC were then intersected with these differential genes. Interestingly, we obtained a hub shared gene *S100A6*, which may be very significant both in T2DM and PC (**Figure 5A** **)**.

The increased expression of S100A6 was firstly validated in 176 PC tissues in comparison to 171 normal pancreatic tissues (p < 0.001) from TCGA and GTEx database (**Figure 5B**). Then, the values of S100A6 expression in 4 tumor samples were considerably higher than that in matching normal samples in TCGA cohort (p = 0.029; **Figure 5C**). In addition, S100A6 was expressed significantly differently in PC tissues with different tumor grades (**Figure 5D**). To further examine S100A6 protein expression, we retrieved representative immunohistochemistry (IHC) stainings from Human protein atlas (HPA) and showed that S100A6 was remarkably overexpressed in PC samples compared to normal tissue. (**Figure 5E**). We also performed an investigation on the prognostic outcomes of S100A6 in PC patients. Kaplan–Meier (K-M) plot showed that high S100A6 expression was closely associated with poor OS (p = 0.005; **Figure 5F**). Univariate Cox analysis revealed that age, grade, and S100A6 expression were markedly correlated with OS (**Table S1**). Multivariate Cox analysis showed a significant relationship between S100A6 overexpression and low OS [hazard ratio (HR): 1.00, p = 0.0034; **Figure 5G**], indicating that the shared gene *S100A6* may be an independent predictor for patients with PC.

### Gene Set Enrichment Analysis Identifies S100A6-Associated Signaling Pathways

**Figure 6:**
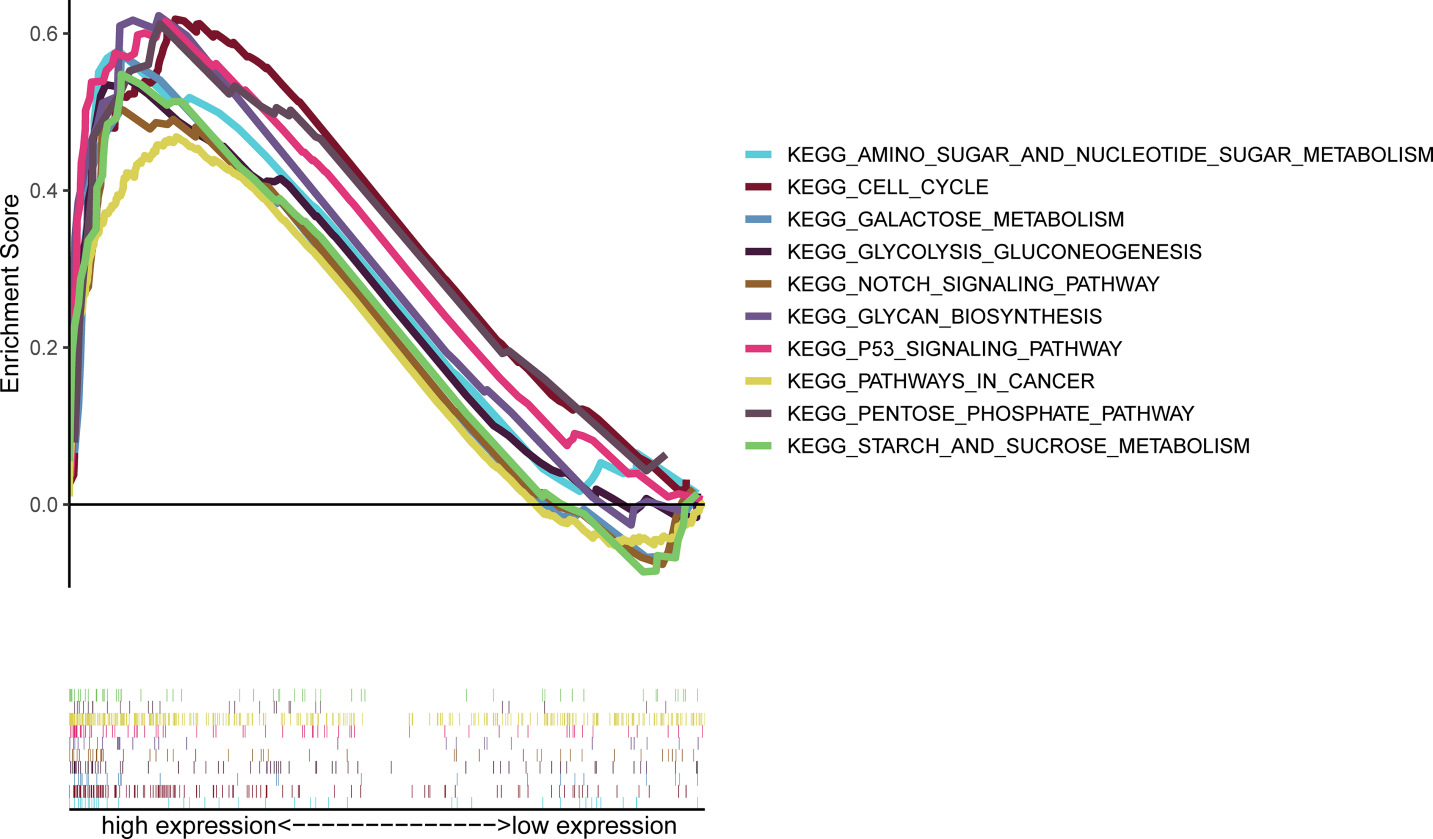
GSEA of the top 10 enriched pathways in PC patients with high S100A6 expression. GSEA, Gene Set Enrichment Analysis; PC, pancreatic cancer.

GSEA results showed that S100A6 overexpression was associated with cancer- and glycometabolism-related pathways such as pathway in cancer, p53 signaling pathway, Notch signaling pathway, and cell cycle, as well as in glycan biosynthesis, pentose phosphate pathway, sugar metabolism, galactose metabolism, and sucrose metabolism, demonstrating that S100A6 was possibly implicated in cancer growth and glucose metabolism (**Figure 6**).

### Association Between S100A6 and the Tumor Microenvironment

**Figure 7:**
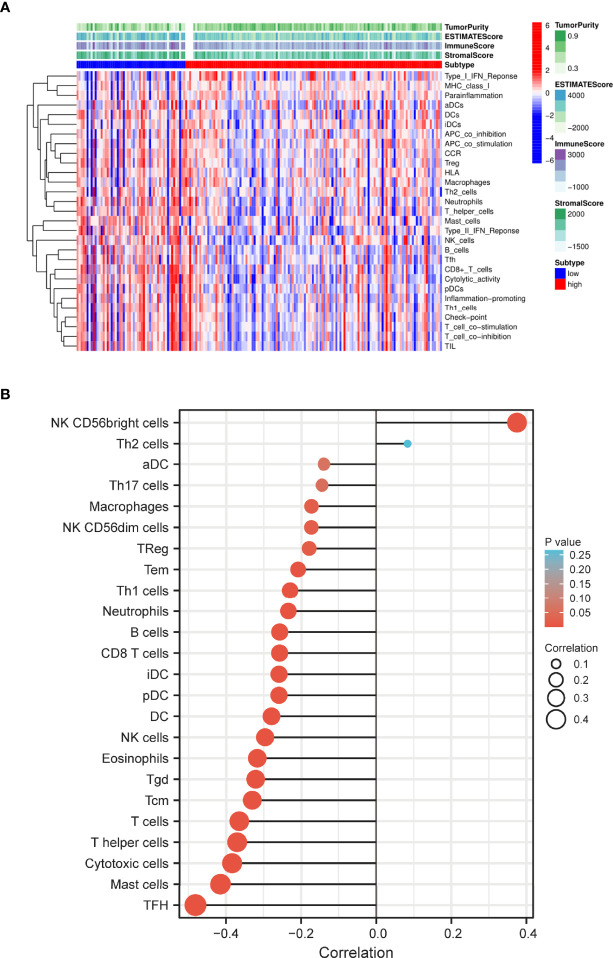
Distribution of immune cell infiltration in PC. **(A)** Heatmap of the immune cells between high and low expression group. **(B)** Relationship of S100A6 expression and immune cell subtypes in PC patients. PC, pancreatic cancer.

Based on the ssGSEA algorithm, we compared TIME landscape between S100A6-high and -low groups (**Figure 7A**). In addition, S100A6 was shown to be positively linked with macrophages, natural killer (NK) cells, regulatory T (Treg), T effective Memory (Tem), T helper 1 (Th1) cells, neutrophils, B cell, clusters of differentiation 8 (CD8) T cell, dentritic cell (DC), eosinophils, T gamma delta (Tgd), T central memory (Tcm), T cells, T helper cells, cytotoxic cells, mast cells, and T follicular helper (TFH) while being negatively correlated with NK CD56bright cells (**Figure 7B**).

**Figure 8:**
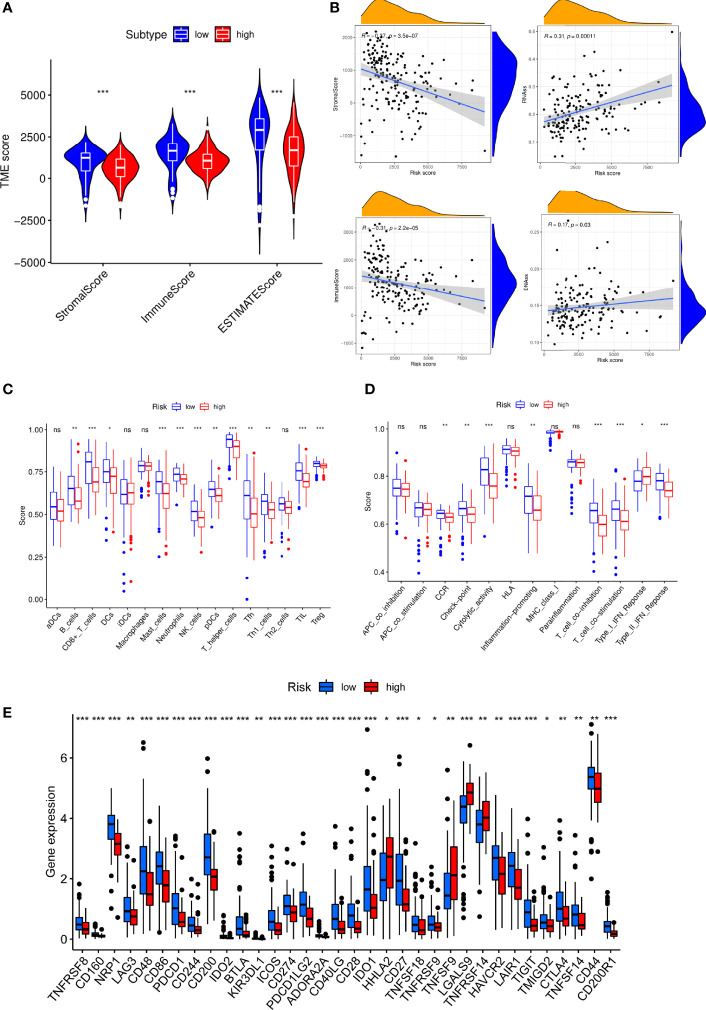
Immune microenvironment analysis in PC patients with high and low S100A6 expression. **(A)** Comparison of ESTIMATE Score, Stromal score, and Immune score between S100A6-high and S100A6-low groups by ssGSEA algorithm. **(B)** Relationship of S100A6 expression and RNAss, DNAss, Stromal Score, and Immune Score. **(C)** Comparison of immune cell subsets, **(D)** immune-related functions, **(E)** immune checkpoints between S100A6-high and S100A6-low groups. PC, pancreatic cancer; ssGSEA, single-sample Gene Set Enrichment Analysis. ns, no significance, ^∗^p < 0.05, ^∗∗^p < 0.01, and ^∗∗∗^p < 0.001.

We discovered that low-S100A6 group had higher EstimateScore, ImmuneScore, and StromalScore levels than those in high-S100A6 group (p < 0.001) (**Figure 8A**). Moreover, S100A6 was significantly negatively correlated with immune score and StromalScore but positively associated with DNAss and RNAss (**Figure 8B**). We also found that Tfh cell, CD8+ T cell, DC cell, and NK cell were increased in low-S100A6 group (**Figures 8C**). The fractions of cytolytic activity, Type II IFN, and T cell function were higher in low-S100A6 group than those in the high group (**Figures 8D**). We further discovered substantial differences in immune checkpoints such as PDCD1, CTLA4, ICOS, and LAG3 between the two groups (**Figure 8E**).

### Inhibition of S100A6 Suppresses Pancreatic Cancer Cell Proliferation and Migration

**Figure 9:**
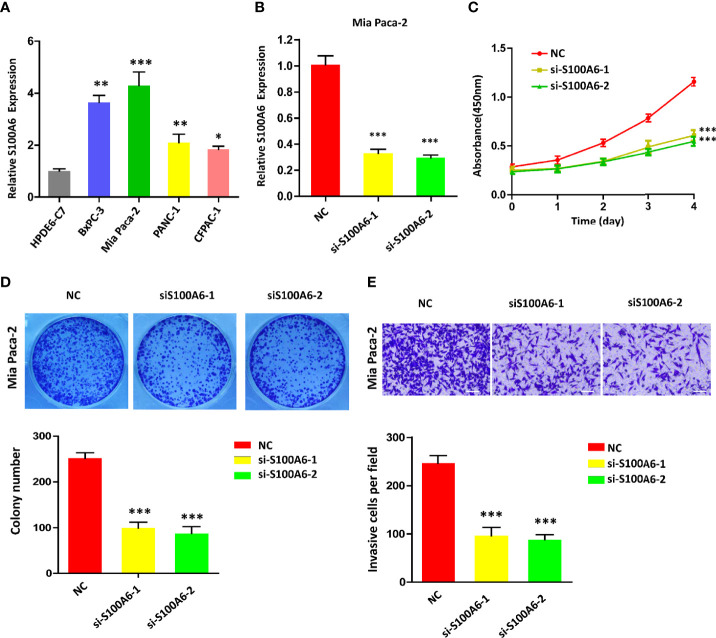
S100A6 knockdown decreases PC cell proliferation, invasion, and migration. **(A)** S100A6 expression levels in five different cells. **(B)** qRT-PCR analysis of S100A6 expression in Mia Paca-2 cell transduced with siRNA. **(C)** CCK-8 of proliferation ability in transduced Mia Paca-2 cell. **(D)** Colony formation assay in transduced Mia Paca-2 cell. **(E)** Transwell assay of migration/invasion ability in transduced Mia Paca-2 cell. PC, pancreatic cancer; qRT-PCR, real‐time quantitative PCR; CCK-8, cell counting kit-8. ^∗^p < 0.05, ^∗∗^p < 0.01, and ^∗∗∗^p < 0.001.

To confirm the value of S100A6 in PC, S100A6 expression levels in five PC cell lines (HPDE6-C7, BxPC-3, Mia Paca-2, PANC-1, CFPAC-1) were examined. Mia Paca-2 cell was found to have the highest S100A6 expression (**Figure 9A**), which was therefore selected for functional analysis. Then, we successfully knocked down the S100A6 expression in Mia Paca-2 cell by si-S100A6 transfection (**Figure 9B**). CCK-8 and colony formation assays showed that S100A6 knockdown cause a decreased proliferation ability of Mia Paca-2 cell (**Figures 9C, D**). Transwell assay showed that inhibiting S100A6 with indicated siRNA resulted in a significant reduction of invaded cells (**Figure 9E**).

## Discussion

Since 1983, Whittemore et al. (24) reported the close relationship between PC and T2DM. As research proceeds, multiple studies have concluded that PC and T2DM are linked in a bidirectional manner. A meta-analysis in 2015 found a 60% increased risk of PC in people with a duration of diabetes ≥2 years compared to those without diabetes and a 50% increased risk in those with ≥10 years (25). Some clinical studies found about 0.85% (18/2122) to 7% (6/86) of T2DM patients were identified with PC within 3 years of disease onset, indicating that T2DM may be a result or early manifestation of PC (26–28). Data from genome-wide association studies (GWAS) revealed that some genes such as *PDX1, HNF1α*, and *UCP2* (29) were involved in the development of T2DM and PC but only explain small portions of heritability and lack a thorough and systemic analysis. However, the molecular mechanism of a complex interaction between PC and T2DM remains unclear. This was the first research to investigate the shared genes and common signatures of PC and T2DM *via* WGCNA to guide earlier detection, better treatment, and timely prevention.

Global gene expression data from pancreas tissues could aid in our better understanding of the interactive pathogenesis of T2DM and PC. The shared genes were enriched highly in biological processes of regulating endodermal cell fate specification, in addition to dendritic cell, IL-1β-related signaling pathway, ion channel activities, timing of anagen, and so on. PPI analysis revealed that these genes were also mainly associated with cell fate decision events such as cell division, cell cycle, mitotic nuclear division, chromosome segregation, DNA replication, transcription, and translation. The pancreas is an endoderm-derived organ that develops through a series of morphological processes to generate distinct cell types, including β-cell, acinar, and ductal cells (30, 31). During pancreas development (32), distinct morphogenesis or cell types could be interconverted. For instance, it is possible for a pancreatic exocrine cell to be transformed to β-cell or endocrine progenitor cell under a certain environment (33). These suggest that specific endodermal lineage might directly affect the potential association between pancreatic-related diseases, such as PC and DM. There are a variety of signaling pathways that participated in coordinating different stages of endoderm development and pancreas organogenesis. It has been shown that Wnt/β-catenin pathway is implicated in endoderm differentiation, and its dysregulation leads to the development of DM (34). Meanwhile, activation of the Wnt pathway could promote PC carcinogenesis (35). TGF-β signaling is another endoderm-specific factor, which could be considered as a link between PC and DM (36, 37). Therefore, we speculate that the common pathogenesis of PC and DM may be because they share common genes and regulators in endodermal fate specification.

To further identify the hub gene, we validate 44 shared genes of PC and T2DM in four validated cohorts by differential gene expression analysis. Interestingly, we found that S100A6 might play an extremely significant role in the progression of PC and T2DM. S100A6 is a calcium-binding protein with EF-hand belonging to the S100 family member. Other S100 members like S100A4, S100A8, S100A9, and S100A16 are structurally similar to S100A6, but their functions are distinct (38). For example, our previous study found that S100A16 mainly participated in lipid metabolism (39, 40). In addition, it has also been found to be involved in several cancers (41), including prostate cancer, lung cancer, and ovarian cancer. Another study indicated that S100A6 could specifically promote calcium-stimulated insulin release, which is associated with hyperglycemia (42). Stancill et al. (43) indicated that S100A6 was remarkedly upregulated in Abcc8^-/-^ β cells, with a 37-fold increase compared to controls. Moreover, *S100A6* gene is located in human chromosome 1q21, where frequent chromosomal rearrangements occur in neoplasia. It has been shown that S100A6 contributes to cell proliferation, migration, and adhesive properties of some cancers, such as breast, stomach, pancreas, and colon cancer (44, 45). Similar to previous reports (46, 47), we also found that S100A6 was significantly overexpressed in PC with a worse prognosis. Further *in vitro* experiments showed that S100A6 promoted cell proliferation, migration, and invasion in PC. Notably, GSEA was enriched not only in cancer-related pathways, including pathway in cancer, p53 signaling pathway, Notch signaling pathway, and cell cycle, but also in a series of glycometabolism-related pathways. The Notch pathway has been recognized as a self-renewal mechanism of endoderm progenitor cells, which is early engaged in pancreas specification, β-cell function, and survival (48, 49). These results suggest that S100A6 may be a common mediator in the PC and T2DM signaling pathways.

Recent research has provided that TIME is an essential element of tumorigenesis and progression (50, 51). In our study, there is a negative correlation between S100A6 expression and numerous immune cell infiltrations in PC patients, such as Tfh cell, cytotoxic cell, and DC cell. Tfh cells are a subgroup of CD4+ T cells that contribute to germinal center (GC) development, antibody maturation, and humoral memory maintenance. Tfh cells could help regulate humoral immunity and antitumor responses in some autoimmune diseases and cancers (52). Studies have shown that infiltrating Tfh cells are protective against breast cancer and colorectal cancer (53, 54). Our findings of Tfh cells in PC support their presence and have been linked to improved survival for patients with low S100A6 expression. In addition, cytotoxic cells, also named CD8+ cytotoxic T cells (CTLs), as well as DC cells are considered to be tumor-antagonizing immune cells. Cancer cells expressing ligands that bind to inhibitory checkpoints can block the activation of CTLs, a key strategy for tumor immune evasion (55). DCs, as antigen-presenting cells (APCs), are capable of presenting antigens and delivering costimulatory signals to activate T lymphocytes. Although DCs are critical for immune activation and effector cell recruitment, tumor cells can block DCs either by avoiding immunological recognition or by disabling effector T cells to escape immune surveillance (56). Meanwhile, the corresponding immune cell markers and immune checkpoints had negative correlations with S100A6 expression. All in all, these results demonstrated that *S100A6*, a shared gene of PC and T2DM, played a significant role in TIME.

However, there are still some limitations to this study. First, the available clinical information in the public database is limited, and the contaminated tissues may cause WGCNA results to be biased. Second, further *in vitro* experiments were needed to better understand the shared mechanism of PC and T2DM “regulation of endodermal cell fate specification.” At last, there is not enough evidence that S100A6 is a good predictor for T2DM and PC patients, and it still needs to be verified in future clinical trials.

**Figure 10:**
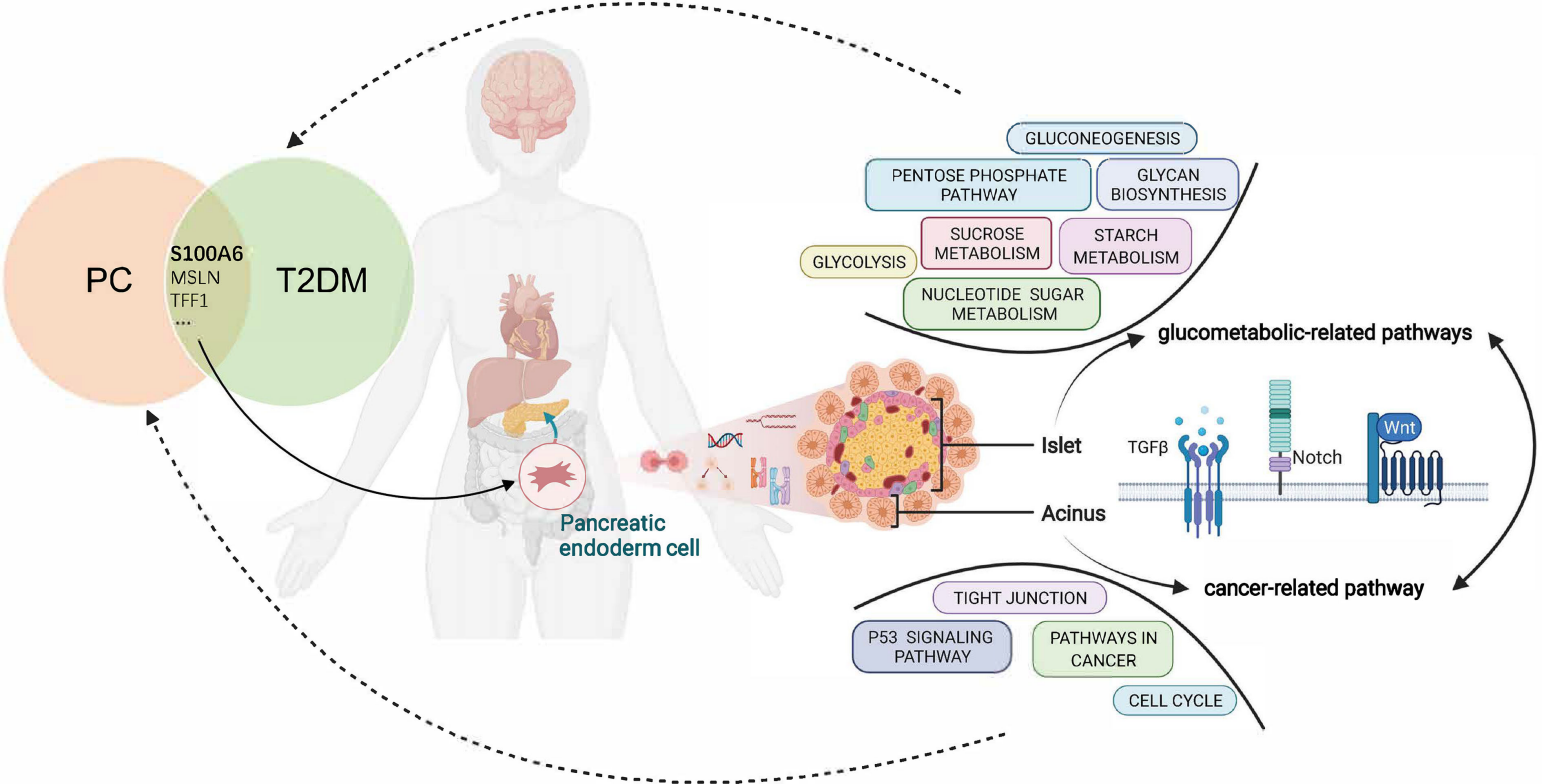
Overview of the interactions between T2DM and PC. The shared genes of T2DM and PC regulate the pancreatic endodermal cell fate and differentiation and are then involved in cancer-related pathways that promote PC development as well as glucometabolic-related pathways that promote T2DM development. T2DM, type 2 diabetes mellitus; PC, pancreatic cancer.

In conclusion, this study proposed the shared gene signatures to illustrate the possible mechanism of PC and T2DM (**Figure 10**), revealed that the regulation of endodermal cell fate specification might be a common pathway, and identified S100A6 as an immune-related biomarker and potential therapeutic target for patients with PC and T2DM.

## Data Availability Statement

The datasets presented in this study can be found in online repositories. The names of the repository/repositories and accession number(s) can be found in the article/**Supplementary Material**.

## Author Contributions

YH, FJ, and YL conceptualized and designed the study. YH and NZ wrote the article. YG and DW collected and analyzed the data. XQ and WZ carried out the experiments. YH, NZ, and YG contributed equally to this work. All authors contributed to the article and approved the submitted version.

## Funding

This work was supported by grants from the National Key Research and Development plan of the Ministry of Science and Technology of China (Grant No. 2018YFC1314900, 2018YFC1314901), the industry prospecting and common key technology key projects of the Jiangsu Province Science and Technology Department (Grant No. BE2020721), the Special guidance funds for service industry of the Jiangsu Province Development and Reform Commission (Grant No. 2019-1089), and the big data industry development pilot demonstration project of the Ministry of Industry and Information Technology of China (Grant No. 2019-243).

## Conflict of Interest

The authors declare that the research was conducted in the absence of any commercial or financial relationships that could be construed as a potential conflict of interest.

## Publisher’s Note

All claims expressed in this article are solely those of the authors and do not necessarily represent those of their affiliated organizations, or those of the publisher, the editors and the reviewers. Any product that may be evaluated in this article, or claim that may be made by its manufacturer, is not guaranteed or endorsed by the publisher.

## Supplementary Material

Supplementary Figure 1 Gene ontology analysis that explores the potential mechanism of the 44 shared genes on the development of type 2 diabetes and pancreatic cancer.

Supplementary Figure 2 Disease ontology enrichment analysis of the shared genes.

Supplementary Table 1 Univariate Cox regression analysis of OS of PC patients in TCGA cohorts.
