# Supplementary material for: Identification of the Shared Gene Signatures and Biological Mechanism in Type 2 Diabetes and Pancreatic Cancer
Source: Front Endocrinol (Lausanne). 2022 Mar 31;13:847760. doi: 10.3389/fendo.2022.847760 (PMC9010232; doi:10.3389/fendo.2022.847760)
Supplement: Supplementary Figure 1 — Gene ontology analysis that explores the potential mechanism of the 44 shared genes on the development of type 2 diabetes and pancreatic cancer. [file DataSheet_1.docx]

Supplemental Tables, Figures and Data

**Table S1. univariate Cox regression analysis of OS of PC patients in TCGA cohorts.**

| **Risk score** | **HR** | **HR.95L** | **HR.95H** | ***P* value** |
| --- | --- | --- | --- | --- |
| Age | 1.028 | 1.006 | 1.050 | 0.011 |
| Gender | 0.897 | 0.588 | 1.368 | 0.614 |
| Grade | 1.377 | 1.020 | 1.859 | 0.036 |
| stage | 1.422 | 0.979 | 2.066 | 0.065 |
| S100A6 | 1.000 | 1.000 | 1.000 | 0.011 |

**Figure S1:**


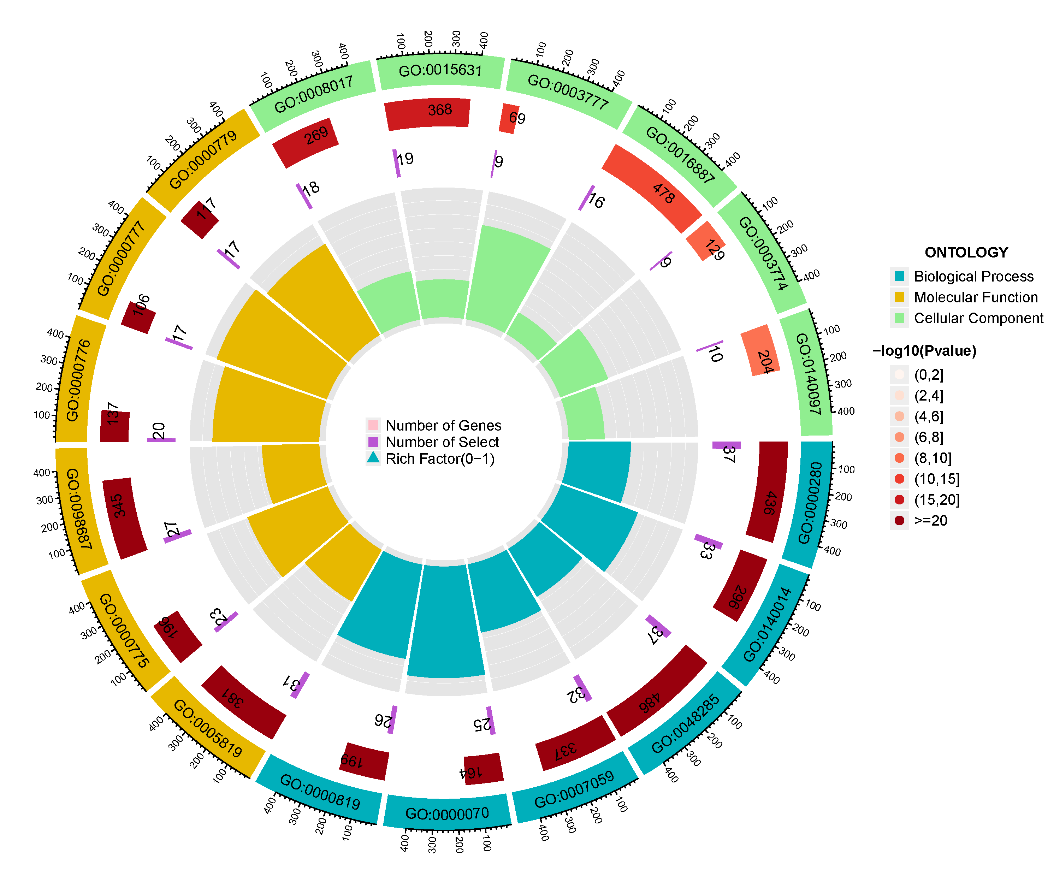


**Figure S1: Gene ontology analysis that explores the potential mechanism of the 44 shared genes on the development of type 2 diabetes and pancreatic cancer.**

**Figure S2:**


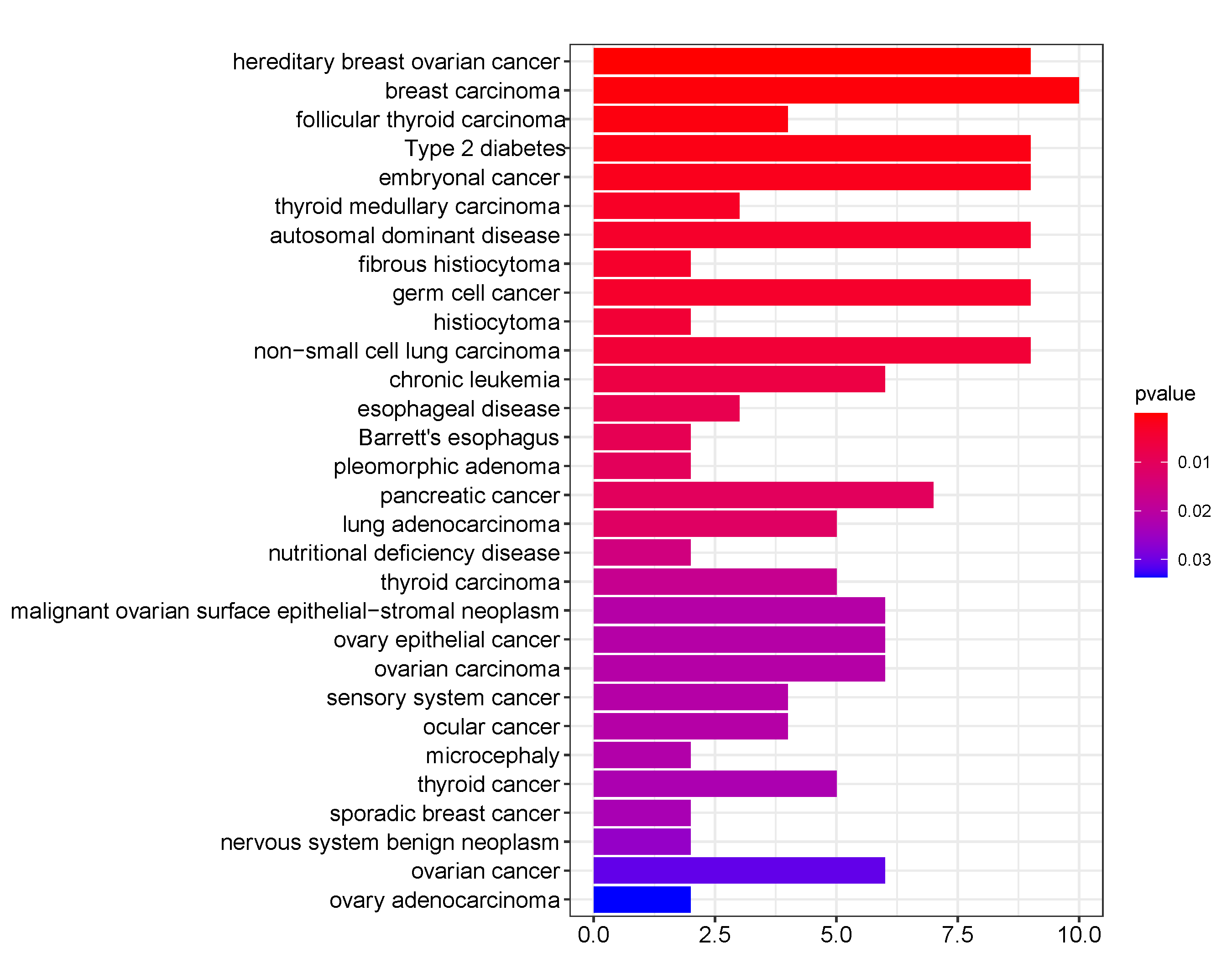


**Figure S2:** **Disease ontology enrichment analysis of the shared genes.**
